# Detection of COVID-19 epidemic outbreak using machine learning

**DOI:** 10.3389/fpubh.2023.1252357

**Published:** 2023-12-18

**Authors:** Giphil Cho, Jeong Rye Park, Yongin Choi, Hyeonjeong Ahn, Hyojung Lee

**Affiliations:** ^1^Department of Artificial Intelligence and Software, Kangwon National University, Samcheok-si, Republic of Korea; ^2^Department of Mathematics, Kyungpook National University, Daegu, Republic of Korea; ^3^Busan Center for Medical Mathematics, National Institute for Mathematical Sciences, Daejeon, Republic of Korea; ^4^Department of Statistics, Kyungpook National University, Daegu, Republic of Korea

**Keywords:** COVID-19, prediction, machine learning, early detection, outbreak

## Abstract

**Background:**

The coronavirus disease (COVID-19) pandemic has spread rapidly across the world, creating an urgent need for predictive models that can help healthcare providers prepare and respond to outbreaks more quickly and effectively, and ultimately improve patient care. Early detection and warning systems are crucial for preventing and controlling epidemic spread.

**Objective:**

In this study, we aimed to propose a machine learning-based method to predict the transmission trend of COVID-19 and a new approach to detect the start time of new outbreaks by analyzing epidemiological data.

**Methods:**

We developed a risk index to measure the change in the transmission trend. We applied machine learning (ML) techniques to predict COVID-19 transmission trends, categorized into three labels: decrease (L0), maintain (L1), and increase (L2). We used Support Vector Machine (SVM), Random Forest (RF), and XGBoost (XGB) as ML models. We employed grid search methods to determine the optimal hyperparameters for these three models. We proposed a new method to detect the start time of new outbreaks based on label 2, which was sustained for at least 14 days (i.e., the duration of maintenance). We compared the performance of different ML models to identify the most accurate approach for outbreak detection. We conducted sensitivity analysis for the duration of maintenance between 7 days and 28 days.

**Results:**

ML methods demonstrated high accuracy (over 94%) in estimating the classification of the transmission trends. Our proposed method successfully predicted the start time of new outbreaks, enabling us to detect a total of seven estimated outbreaks, while there were five reported outbreaks between March 2020 and October 2022 in Korea. It means that our method could detect minor outbreaks. Among the ML models, the RF and XGB classifiers exhibited the highest accuracy in outbreak detection.

**Conclusion:**

The study highlights the strength of our method in accurately predicting the timing of an outbreak using an interpretable and explainable approach. It could provide a standard for predicting the start time of new outbreaks and detecting future transmission trends. This method can contribute to the development of targeted prevention and control measures and enhance resource management during the pandemic.

## 1 Introduction

The coronavirus disease (COVID-19) pandemic is caused by the novel coronavirus SARS-CoV-2, which has spread rapidly and affected human lives worldwide. Since the start of the pandemic non-pharmaceutical interventions (NPIs) such as wearing masks, social distancing, and pharmaceutical vaccination have been implemented to control the spread of the virus. However, the emergence of new variants of the virus has raised concerns about their potential for increased transmission. The pandemic continues to impact human lives, and it is crucial to control it and reduce its transmission.

Predictions can be made in several ways. One common approach is to use mathematical models that consider factors such as the rate of transmission, number of cases, and effectiveness of control interventions such as social distancing and vaccination. These models can predict future trends in COVID-19 transmission dynamics and estimate the number of cases and deaths (1–3). Mathematical models are widely used for predicting infectious diseases, but they can be difficult to adapt to various external factors such as social distancing or the emergence of new variants (4, 5).

Another approach is to use machine learning (ML) methods to detect changes in the trend of transmission and potential outbreaks (6–9). Shahid et al. (6) predicted the confirmed cases, deaths, and recoveries of COVID-19 in 10 major countries using ARIMA, SVR, LSTM, and Bi-LSTM. Chakraborty et al. (10) performed short-term forecasts of future COVID-19 cases in Canada, France, Republic of Korea, the United Kingdom, and India, using a hybrid forecasting approach based on the ARIMA and wavelet-based models. Katragadda et al. (9) explored the COVID-19 spread growth in America by comparing the mobility of local people and visitors, and forecasted the number of cases using various ML models.

Investigating the start point of infectious disease outbreaks and analyzing the transmission dynamics of epidemics is critical for several reasons. First, understanding the source of an outbreak can help identify the underlying cause of the disease and prevent future outbreaks. Second, analyzing the transmission dynamics of epidemics can provide important information on how the disease spreads and who is at risk. This information can then be used to develop effective preventive and control measures. Third, investigating the start point of an outbreak and analyzing the transmission dynamics can help determine the scope and severity of the outbreak. This information is important for determining the level of response required to control an outbreak and to protect public health. Therefore, understanding the start point of infectious disease outbreaks and analyzing transmission dynamics is essential for the effective investigation, prevention, and control of outbreaks.

Early detection (ED) methods and warning systems for epidemics are important to prevent and control the spread of the virus. Shi et al. (11) developed statistical models combining least absolute shrinkage and selection operator with the ARIMA model to forecast the spread of dengue pandemic in Singapore. Several studies have used statistical methods for the ED of infectious disease outbreaks using statistical methods (11–13). ML has been proposed as a useful tool for ED of COVID-19 outbreak (14–16). Martinez-Velazquez et al. (14) detected the COVID-19 outbreak using self-reported symptom data and evaluated the performance of models using 15 ML classifiers, such as decision tree, neural network, Support Vector Machine (SVM), and Random Forest (RF).

Korea experienced five reported outbreaks from March 2020 to October 2022. The start times of outbreaks were not clearly determined, as different start dates were reported, as summarized in Supplementary Table S1. Here, we investigated national COVID-19 outbreaks without considering regional factors, as the country’s size is not very large (17). Additionally, policy decisions related to COVID-19 are managed at the national level by the Korea Disease Control and Prevention Agency (KDCA). No explainable standards were recommended to determine the start time of the COVID-19 outbreak. In this study, we aimed to develop a method to detect early COVID-19 outbreaks or identify potential early outbreaks using ML by analyzing epidemiological data in the Republic of Korea.

## 2 Methods

**Figure 1:**
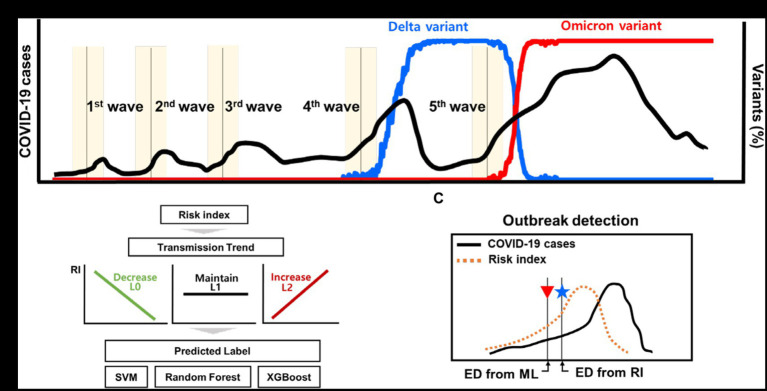
Schematic for the outbreak detection of COVID-19 outbreak. **(A)** The reported dates of the new COVID-19 outbreaks and the proportion of variants. **(B)** Transmission trend is estimated using ML techniques of classification. **(C)** Detection of new outbreak using the risk index and ML techniques.

The method used to detect the emergence of the COVID-19 outbreak is illustrated in Figure 1. We propose a novel method using the risk index and machine learning, without requiring any new developments in the machine learning method. This approach enables us to interpret the transmission trend using the risk index function and various data.

### 2.1 Epidemiological data

We analyzed epidemiological data on reported cases of COVID-19 from February 18, 2020 to October 31, 2022, provided by KDCA (18) in the Republic of Korea, shown in Supplementary Figures S1A,B. The proportions of delta and omicron variants were obtained from covariance data (19, 20). We computed the number of delta variant cases and omicron cases by multiplying the daily COVID-19 cases with proportional data (18–20).

Previous studies mentioned that enhanced social distancing was a crucial intervention to prevent the spread of COVID-19 transmission in Korea (21–23).

We used collected data on social distancing measures among NPIs from a press release by KDCA (24), where we divided the levels of social distancing into four categories based on their intensity (distancing level 1 to 4) (25–27). Supplementary Table S2 summarizes the important times to change the level of social distancing. The higher the level, the more stringent the control intervention implemented. In addition, Supplementary Figure S1C and Supplementary Table S3 show the proportion of days of the week on the yearly number of COVID-19 cases.

### 2.2 Ethical considerations

The data are presented in Supplementary Table S3. The datasets were fully anonymized and did not include any personally identifiable information. Thus, ethical approval was not required for this analysis.

### 2.3 Overview of the estimation of transmission trend of COVID-19 epidemic

Figure 1 shows a schematic of the detection of early outbreaks. Figure 1A shows newly reported COVID-19 cases and several outbreaks in Korea, along with the proportion of variants. Figures 1B,C shows a new method for estimating the start time of the new outbreak.

### 2.4 Sample data

#### 2.4.1 Define calibration and prediction periods

**Figure 2:**
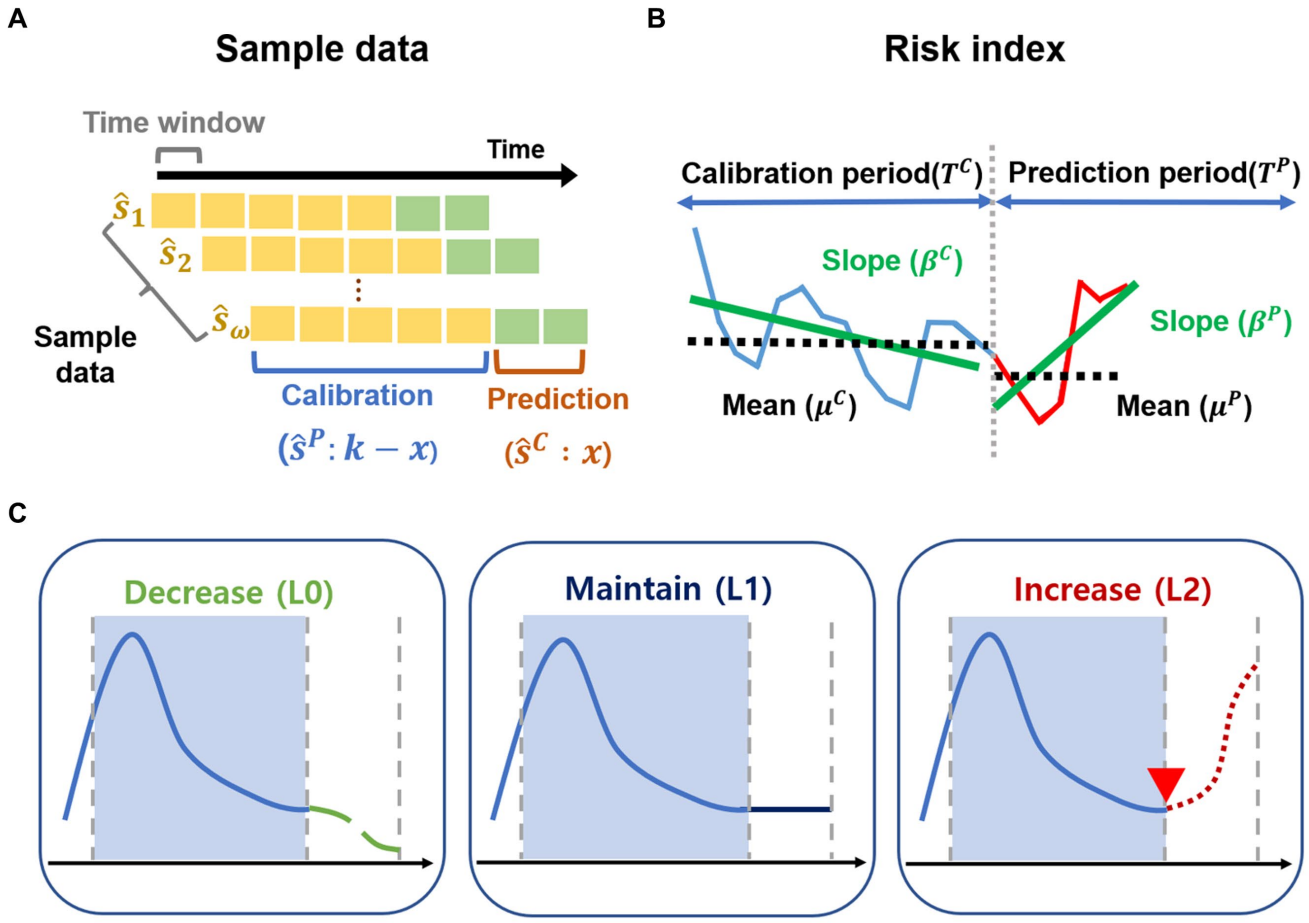
Sample data and risk index. **(A–C)** Outline of the methods. **(A)** The sample data are generated for the calibration period and prediction period from February 2020 to October 2022. **(B)** Risk index for transmission trend is developed. **(C)** Transmission trends are grouped as decrease (L0), maintain (L1), increase (L2) using risk index.

The daily number of COVID-19 cases was collected for specific periods of *k* days. Let $I\left(t\right)$ denote the number of COVID-19 cases on day *t*. The first sample data of the cases is defined as $s_{1}=\left\{I_{1}\left(1\right),I_{1}\left(2\right),\ldots ,I_{1}\left(k\right)\right\}$ , where $I_{\omega }\left(t\right)$ denotes $I\left(t\right)$ on the ω -th sample data. The sample data comprise two partitions of time periods: a calibration period, excluding the most recent *x* days, and a prediction period, including the most recent x days to predict the most recent *x* days, where the length of the calibration period is y=k−x and the length of the prediction period is *x*, as shown in Figure 2A.

In other words, the sample data $s_{1}$ can be expressed as $s_{1}=s_{1}^{C}\cup s_{1}^{P}\text{,}$ where $s_{1}^{C}=\left\{I_{1}\left(1\right),I_{1}\left(2\right),\ldots ,I_{1}\left(y-1\right),I_{1}\left(y\right)\right\}$ denotes the sample data for the calibration period and $s_{1}^{P}=\left\{I_{1}\left(y+1\right),\ldots ,I_{1}\left(k\right)\right\}$ denotes the sample data for the prediction period. In general, for the time window $\omega \in \left\{1,\ldots ,n\right\}$ with a total of *n* sample data, the ω -th sample data of the cases are defined as $s_{\omega }=\left\{I_{\omega }\left(\omega \right),I_{\omega }\left(1+\omega \right),\ldots ,I_{\omega }\left(k-1+\omega \right)\right\}$ .

The time interval for each ω -th sample data is defined as $T_{\omega }=\left\{\omega ,1+\omega ,\ldots ,k-1+\omega \;\right\}$ . $T_{\omega }$ comprises the time period for the calibration period ( $T_{\omega }^{C}$ ) and the time period for the prediction period ( $T_{\omega }^{P}$ ), expressed by $T_{\omega }=T_{\omega }^{C}\cup T_{\omega }^{P}$ , where the time periods are defined as $T_{\omega }^{C}=\left\{\omega ,1+\omega ,\ldots ,\tau_{\omega }\right\}$ and $T_{\omega }^{P}=\left\{\tau_{\omega }+1,\ldots ,k-1+\omega \right\}$ , and $\tau_{\omega }=\omega -1+y$ is the final time of the calibration period.

Moreover, for each ω -th sample data, $\mu_{\omega }^{C}$ and $\sigma_{\omega }^{C}$ denote the mean and standard deviation of $s_{\omega }^{C}$ for the calibration period, respectively. Likewise, $\mu_{\omega }^{P}\;\mathrm{and}\;\sigma_{\omega }^{P}$ are the average number and standard deviation of $s_{\omega }^{P}$ for the prediction period, respectively.

In the present study, we set the calibration period to 21 days (i.e., k=35,x=14 ) and the time window as 1 day from February 18, 2020, to October 31, 2022. The sample data of the cases consisted of 953 sets (i.e., n= 953), which comprised 667 training data and 286 test data (the ratio of train data to test data was assumed to be 7:3), where all sample data of the cases were defined as $S=\left\{s_{1},s_{2},\ldots ,s_{953}\right\}$ . We considered various periods, where the calibration periods ranged from 14 to 28 days and the predication periods ranged from 7 to 21 days, assuming that the calibration periods were longer than the prediction periods.

#### 2.4.2 Normalization and regression analysis

We normalized the sample data from $s_{\omega }$ to ${\hat{s}}_{\omega }$ using the min-max normalization. Moreover, we applied the linear regression model to the sample data for the calibration period ( ${\hat{s}}_{\omega }^{C}$ ) and prediction period ( ${\hat{s}}_{\omega }^{P}$ ), where ${\hat{s}}_{\omega }={\hat{s}}_{\omega }^{C}\cup {\hat{s}}_{\omega }^{P}$ . Here, $\beta_{\omega }^{C}$ and $\beta_{\omega }^{P}$ denote the slopes obtained from the linear regression model for the samples ${\hat{s}}_{\omega }^{C}$ and ${\hat{s}}_{\omega }^{P}$ , respectively, which are defined as the increment rates. $\mu^{C}=\left\{\mu_{\omega }^{C}\right\}$ denotes the vector of the mean number of COVID-19 cases during the calibration period. $\mu^{P}=\left\{\mu_{\omega }^{P}\right\}$ denotes the vector of the average number of COVID-19 cases during the prediction period. That is, the regression analysis for each sample data ω as follows:

$$\left\{ \begin{array}{c} {\hat{s}}_{\omega }^{C}=\alpha_{\omega }^{C}+\beta_{\omega }^{C}t,t\in T_{\omega }^{C}\\ {\hat{s}}_{\omega }^{P}=\alpha_{\omega }^{P}+\beta_{\omega }^{P}t,t\in T_{\omega }^{P} \end{array}\right.$$

where $\alpha_{\omega }^{C},\alpha_{\omega }^{P}$ are intercept values of the linear regression model for calibration period and prediction period, respectively. $\sigma^{C}=\left\{\sigma_{\omega }^{C}\right\}$ denotes the vector of the standard deviation of the COVID-19 cases for the calibration period. $\sigma^{P}=\left\{\sigma_{\omega }^{P}\right\}$ denotes the vector of the standard deviation of COVID-19 cases for the prediction period. “*Week*” represents the day of the week, corresponding to final time of the calibration period ( $\tau_{\omega })$ . “*Delta*” denotes the number of delta variant and “*Omicron*” denotes the number of omicron variant. “*Policy*” denotes the level of NPIs implemented in Korea.

### 2.5 Development of risk index and labeling for transmission trend

In the present study, we developed a method for early detection of potential infectious disease outbreaks by estimating the starting point of such outbreaks. Previous studies have focused on detecting outbreaks early through statistical or machine learning techniques based on data such as the number of COVID-19 cases, NPIs, and variant viruses in (11–16). As an alternative new approach, we aimed to quantify the risk potential to indicate the increasing trends and changes of transmission trends from calibration period to prediction period.

#### 2.5.1 Definition of risk index

We proposed a quantitative representation of these changes as the risk index, which can be used to classify the risk of potential outbreaks, as described in Figure 2B. For each ω -th sample data, we selected two functions of f and g for transmission trend changes, which consist of the mean of COVID-19 cases ( $\mu_{\omega }^{C}$ , $\mu_{\omega }^{P}$ ) and the increment rate ( $\beta_{\omega }^{C}$ , $\beta_{\omega }^{P}$ ) for calibration period and prediction period, respectively. $c_{1}\;\mathrm{and}\;c_{2}$ represent the positive scaling parameters of the functions f and g . The risk index $\left[RI\left(\tau_{\omega }\right)\right]$ is expressed as follows.

$$RI\left(\tau_{\omega }\right)=f\left(\omega \right)\;g\left(\omega \right)=\sinh\left(c_{1}\left(\frac{\mu_{\omega }^{P}-\mu_{\omega }^{C}}{\mu_{\omega }^{C}}\right)\right)e^{c_{2}\left(\beta_{\omega }^{P}-\beta_{\omega }^{C}\right)}\text{.}$$

(1)

- **Change of the mean of COVID-19 cases**: The function f represents the rate of change to describe how much the COVID-19 cases have increased during the prediction period based on the calibration period. The function f denotes the hyperbolic sine (sinh) function of relative difference between $\mu_{\omega }^{P}$ and $\mu_{\omega }^{C}$ divided by $\mu_{\omega }^{C}$ . If $\mu_{\omega }^{P}>\mu_{\omega }^{C}$ , the function f exhibits positive exponential growth. Otherwise, the function f becomes negative exponential decay.
- **Change of the increment rate of COVID-19 cases**: The function g represents the change of the increment rate for transmission trend to describe how much the slope in prediction period ( $\beta_{\omega }^{P}$ ) has increased from the slope in calibration period ( $\beta_{\omega }^{C}$ ) for the linear regression model. The function g is defined as an exponential function of the difference between $\beta_{\omega }^{P}$ and $\beta_{\omega }^{C}$ . If $\beta_{\omega }^{P}>\beta_{\omega }^{C}$ , the function g has positive exponential growth with g>1 . Otherwise, the function g becomes exponential decay with 0<g≤1 .

We defined the risk index as the product of two functions. For example, one sample shows $\mu_{\omega }^{P}>\mu_{\omega }^{C}$ and $\beta_{\omega }^{P}>\beta_{\omega }^{C}$ . Then, the function f exhibits positive exponential growth. The function g amplifies the function f because of g>1 . However, another sample shows $\mu_{\omega }^{P}>\mu_{\omega }^{C}$ and $\beta_{\omega }^{P}<\beta_{\omega }^{C}$ . Then, the function f exhibits positive exponential growth. The function g plays a role in decreasing the function f because of 0<g≤1 .

#### 2.5.2 Labeling for transmission dynamics using risk index

We calculated the values of risk index for each sample data point ( $S=\left\{s_{1},s_{2},\ldots ,s_{953}\right\}$ ). We uniformly divided the values of risk index ${\left\{RI\left(\tau_{\omega }\right)\right\}}_{\omega \in \left\{1,\ldots ,n\right\}}$ into three groups and determined labels as decrease (L0), maintain (L1), and increase (L2) in the transmission trend. We used a dataset with a similar size for each class (or label) as demonstrated in the previous study (28).

For instance, in the groups with small values of risk index, $RI\left(\tau_{\omega }\right)$ , indicating L0, we interpreted that the transmission trend would decrease for the prediction period, compared to that in the calibration period. Supplementary Figures S2A–C shows examples of the sample data labeled in L0, L1, and L2, respectively.

### 2.6 Machine learning approaches to estimate the transmission trend

We used eight features to estimate the transmission trends using ML techniques. Table 1 summarizes the features of the training and testing sample data.

**Table 1:** Description of features for training the sample data.

Features	Description
$\mu^{C}$	Average number of COVID-19 cases for calibration period
$\sigma^{C}$	Standard deviation of COVID-19 cases for calibration period
$\beta^{C}$	Slope obtained from the linear regression model of COVID-19 cases for calibration period
Week	Start day of the week for calibration period
$\mathrm{Delt}a^{C}$	Average number of Delta variant for calibration period
$\mathrm{Omicro}n^{C}$	Average number of Omicron variant for calibration period
$\mathrm{Polic}y^{C}$	Average level of NPIs for calibration period
$\mathrm{Polic}y^{P}$	Average level of NPIs for prediction period

We applied ML techniques such as SVM, RF, and XGB (29–31). SVM is a supervised learning ML model used for classification. SVM uses support vectors to define decision boundaries and classifies unclassified points by comparing them with the corresponding decision boundaries.

SVM can be considered a model that adds a constraint condition to the perceptron-based model to find the most stable decision boundary. RF is a type of ensemble learning method used for classification and regression. It learns multiple decision trees in parallel to output classification or average predictions. A feature of RF is that the trees have slightly different characteristics due to their randomness. This property results in the decorrelation of the predictions of each tree, thereby improving the generalization performance. In addition, randomization makes the forest robust to noise data. XGB is an ensemble model that uses the boosting technique in a number of decision trees, which represents Extreme Gradient Boosting. XGB is characterized by the implementation of parallel learning to support Gradient Boost, an algorithm implemented using the existing boosting technique. In addition, XGB has a strong resistance to overfitting owing to its regularization function.

Grid search methods were used to determine the best performing hyperparameters for the three models. We used a 10-fold cross validation of the training data to determine the best performance. As a result of applying the grid search method to the three ML methods, the regularization parameter, gamma, and kernel in SVM were 50, 0.3, and the radial basis function, respectively. The number of trees and maximum depth of the RF and XGB algorithms were 85 and 14, and 110 and 7, respectively. Supplementary Table S4 summarizes the range of parameters used in the grid search process. We divided the training and test data into the same ratio for label 0, label 1, and label 2. To evaluate the performance of the three models, we show confusion matrices and receiver operating characteristic (ROC) curves for the test data and compare the accuracy of the three models with *F*1-score and AUC for L0, L1, and L2. We used Python language version 3.10 and scikit-learn version 1.1.3. In addition, we used *SVC*, *RandomForestClassifier*, *XGBClassifier* functions of scikit-learn to simulate the three classification algorithms.

### 2.7 Outbreak detection method

Determining the start time of the new outbreak is important for controlling the spread of COVID-19. Supplementary Table S5 lists the start time of the reported outbreaks in Korea, including the important characteristics of each outbreak. In this study, we propose a new approach to detect a new outbreak, which we called as “estimated outbreak,” described in Figure 2C. We compared the reported outbreaks with the estimated outbreaks.

Estimated outbreaks have two approaches. First, we determined the estimated outbreak using the risk index. We defined the start time of the new outbreak as the first day when L2 designated from risk index (RI) was maintained for at least 14 days. The start time of the early outbreak estimated from RI is denoted by ED from RI. Second, we determined the estimated outbreak using the machine learning methods. We defined the start time of the new outbreak as the first day when label 2, estimated from ML methods, was maintained for at least 14 days, denoted by ED from ML. There are three ED from ML methods; (i) ED from SVM, (ii) ED from RF, and (iii) ED from XGB. Here, 14 days is the duration of the maintenance. Republic Korea’s COVID-19 prevention policy is established after more than 2 weeks, which is why we designated a 2 weeks period. We varied the duration of maintenance between 7–28 days.

Moreover, we analyzed the performance of the proposed methods around ED from RI. To do that, we compared the start time of estimated outbreaks during the 4 weeks, 2 weeks before and after the ED from RI. We defined and set the warning period and the interval for comparing the performance of the ML methods to be 4 weeks.

### 2.8 Data availability

We developed the proposed method in Python 3.10 and made the codes using source data freely available on GitHub at https://github.com/modeling-computation/covid-19_outbreak/.

## 3 Results

### 3.1 Estimation of the transmission trend

Supplementary Figure S2 shows examples of the sample data with three labels. We calculated the correlation between the labels and the scaling parameters in Eq. (1). The labels, which were classified using the risk index, accurately reflected the trend of increase, maintenance, and decrease in Supplementary Figure S3. We set the scaling parameters to 0.01 because the correlation was high (0.6) when $c_{1}$ and $c_{2}$ were 0.01, as Supplementary Figure S3A shows. Supplementary Figure S3B displays the correlations between the labels and all eight features described in Table 1. The slope ( $\beta^{C}$ ) and standard deviation of the COVID-19 cases ( $\sigma^{C}$ ) for the calibration period had a strong correlation with labels. Supplementary Figure S3C illustrates the range of the risk index for each label using a box plot. The box plot clearly indicates that high values of the risk index correspond to label 2.

**Figure 3:**
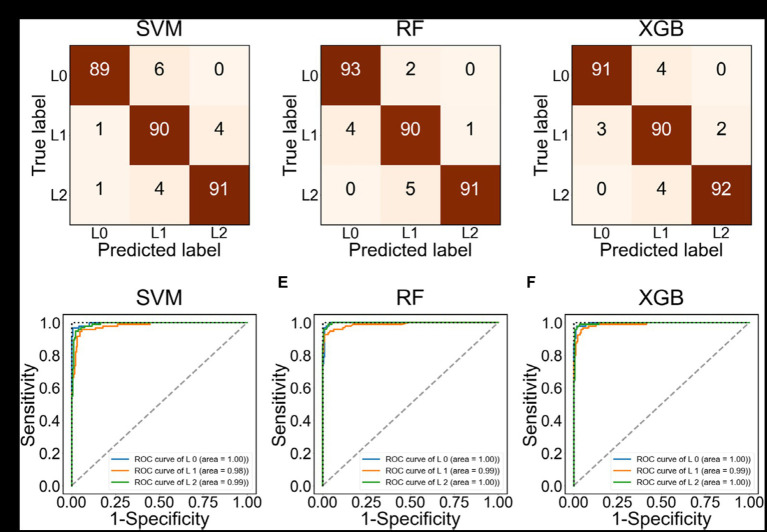
Confusion matrix and ROC curve using the test data labeled as L0, L1, L2. **(A–C)** Confusion matrix using SVM, RF, and XGB, respectively. **(D–F)** ROC using SVM, RF, and XGB, respectively.

Figure 3 evaluates the performance of ML methods such as SVM, RF, and XGB. Figures 3A–C presents confusion matrices for each method. The most critical errors occur when either predicting L2 when the actual label is L0, or predicting the L0 when the actual label is L2. RF and XGB did not make any of these errors, while SVM had two such cases. Figures 3D–F depicts the ROC curve for each class. The area under the curve (AUC), which measures accuracy in the ROC curve, was found to be close to 1 for all three ML methods. Table 2 summarizes the accuracy of the ML methods. The accuracies of SVM, RF, and XGB were higher than 0.94, with values of 0.9441, 0.9580, and 0.9545, respectively. The prediction of the *F*1-score for L0 (Decrease) or L2 (Increase) was particularly accurate, with values of 0.95 and higher.

**Table 2:** Accuracy of test data in three ML methods.

Estimator	Accuracy	*F*1-score		
		Label 0 (L0: decrease)	Label 1 (L1: maintain)	Label 2 (L2: increase)
SVM	0.9441	0.9570	0.9231	0.9529
RF	0.9580	0.9688	0.9375	0.9681
XGB	0.9545	0.9630	0.9326	0.9684

**Figure 4:**
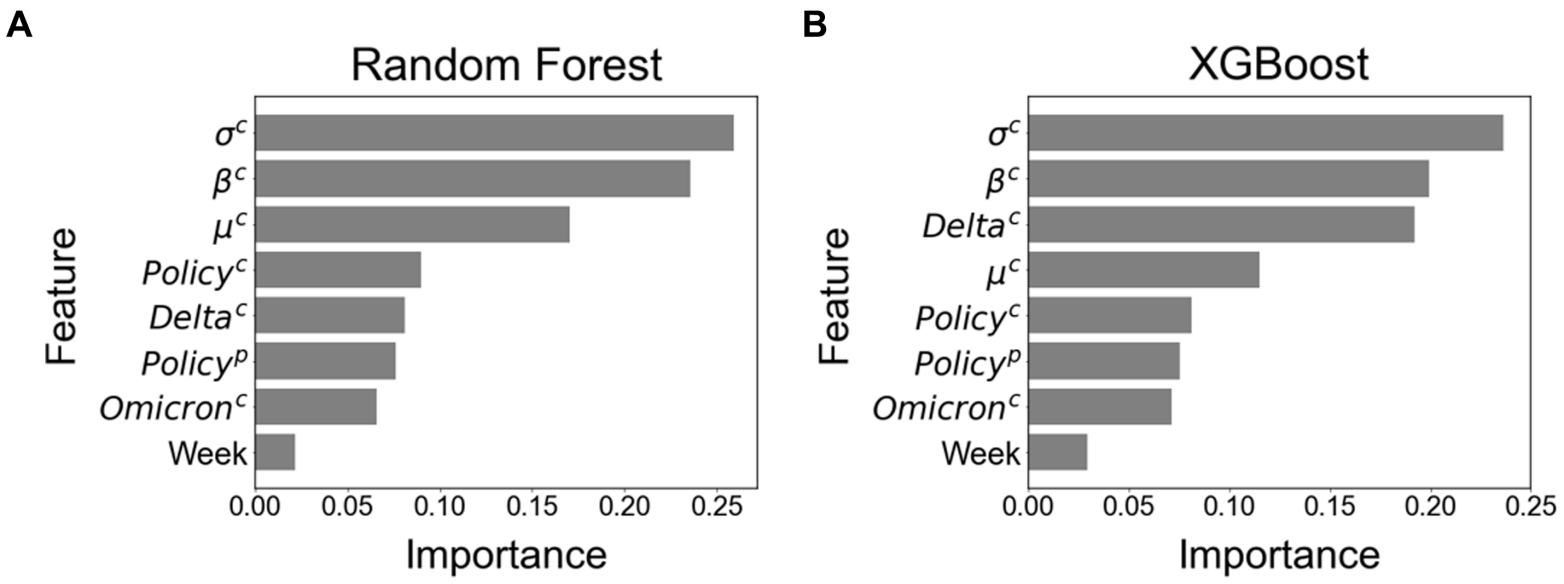
Feature importance among all eight features. **(A)** Feature importance using Random Forest. **(B)** Feature importance using XGBoost.

Figure 4 shows the feature importance in RF and XGB. The features of standard deviation ( $\sigma^{C}$ ), the increment rate ( $\beta^{C}$ ), and mean ( $\mu^{C}$ ) of the COVID-19 cases for the calibration period were important for both methods. The control intervention ( $\mathrm{Polic}y^{C}$ ) also had a high rank of importance in RF, and the delta variant ( $\mathrm{Delt}a^{C}$ ) was an important feture in XGB.

We conducted a sensitivity analysis by changing the calibration period from 14 to 28 days and the prediction periods from 7 to 21 days, as Supplementary Table S6 indicates. The results showed that the highest accuracy was achieved with a calibration period of 21 days and a prediction periods of 14 days.

### 3.2 Estimation of the start time for outbreaks

**Figure 5:**
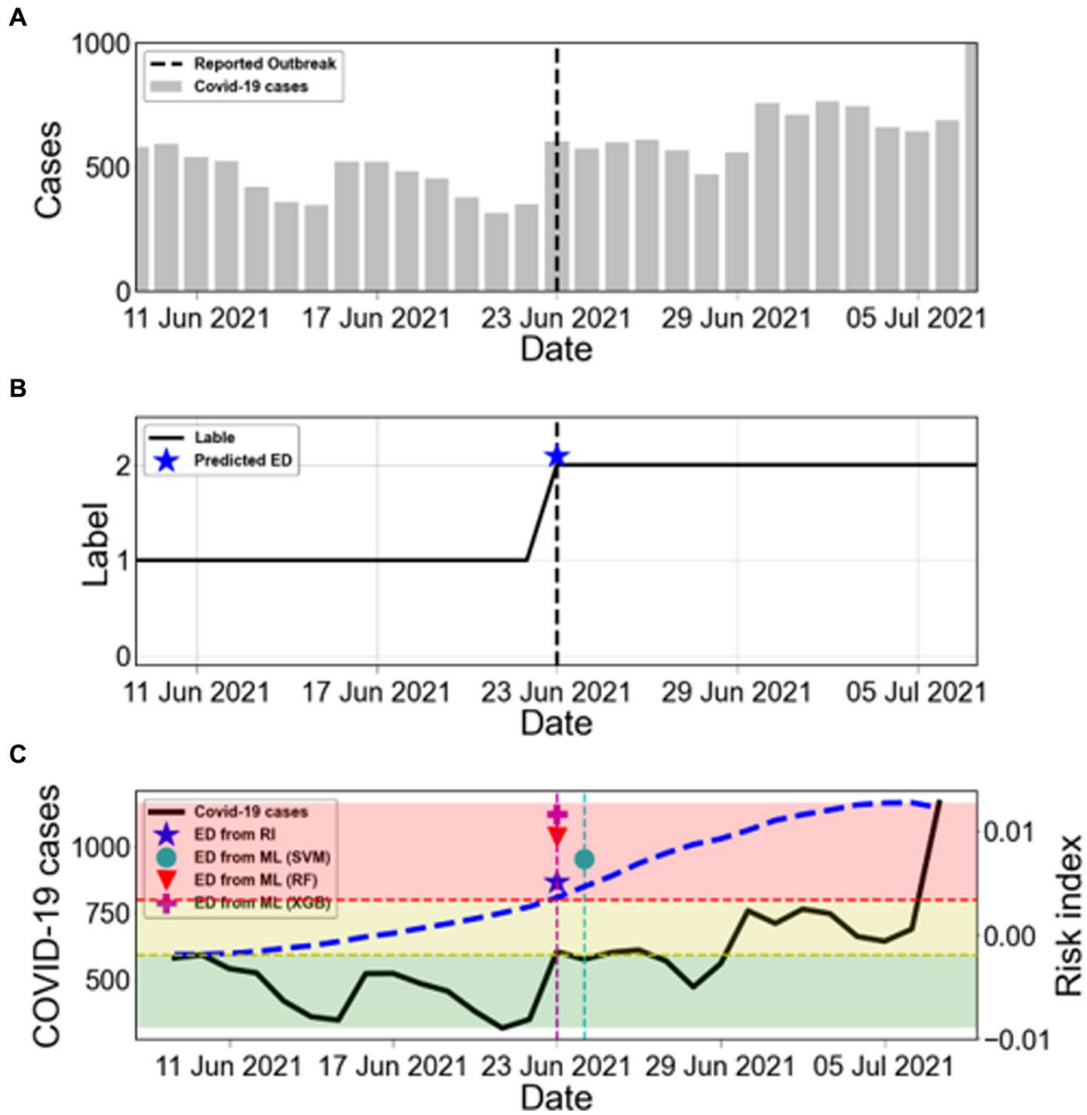
Estimation of the start time of COVID-19 outbreaks. **(A)** The bars show the COVID-19 cases from 9 June 2021 to 7 July 2021. The black dashed line marks the reported outbreak. **(B)** The label is obtained from the risk index. The blue asterisk (★) represents ED from RI. **(C)** Comparison between ED from RI and ED from ML during the warning period from ED from RI. The black solid line shows the number of COVID-19 cases (left *y*-axis). The blue dashed line shows the calculated risk index (RI) (right *y*-axis). The results of ED from ML are marked as SVM (●), RF (▼), and XGB (+). The shaded areas indicate the labels as L0 (green), L1 (yellow), and L2 (red) according to the risk index.

Korea experienced several outbreaks between March 2020 and October 2022. Figure 5A shows the number of COVID-19 cases from 9 June 2021 to 7 July 2021 for an estimated outbreak. The black dashed line in Figure 5A represents the reported outbreak. The asterisks in Figure 5B (★) presents the ED from RI. The shaded areas indicate the labels as L0 (green), L1 (yellow), and L2 (red) according to the risk index. We determined the start time of the new outbreak when the label remained at L2 for 2 weeks, which was the duration of maintenance. Therefore, the ED from RI for this outbreak was 23 June 2021. Figure 5C compares the ED from RI with the ED from ML. The ED from RF and ED from XGB showed the same dates as the ED from RI.

**Figure 6:**
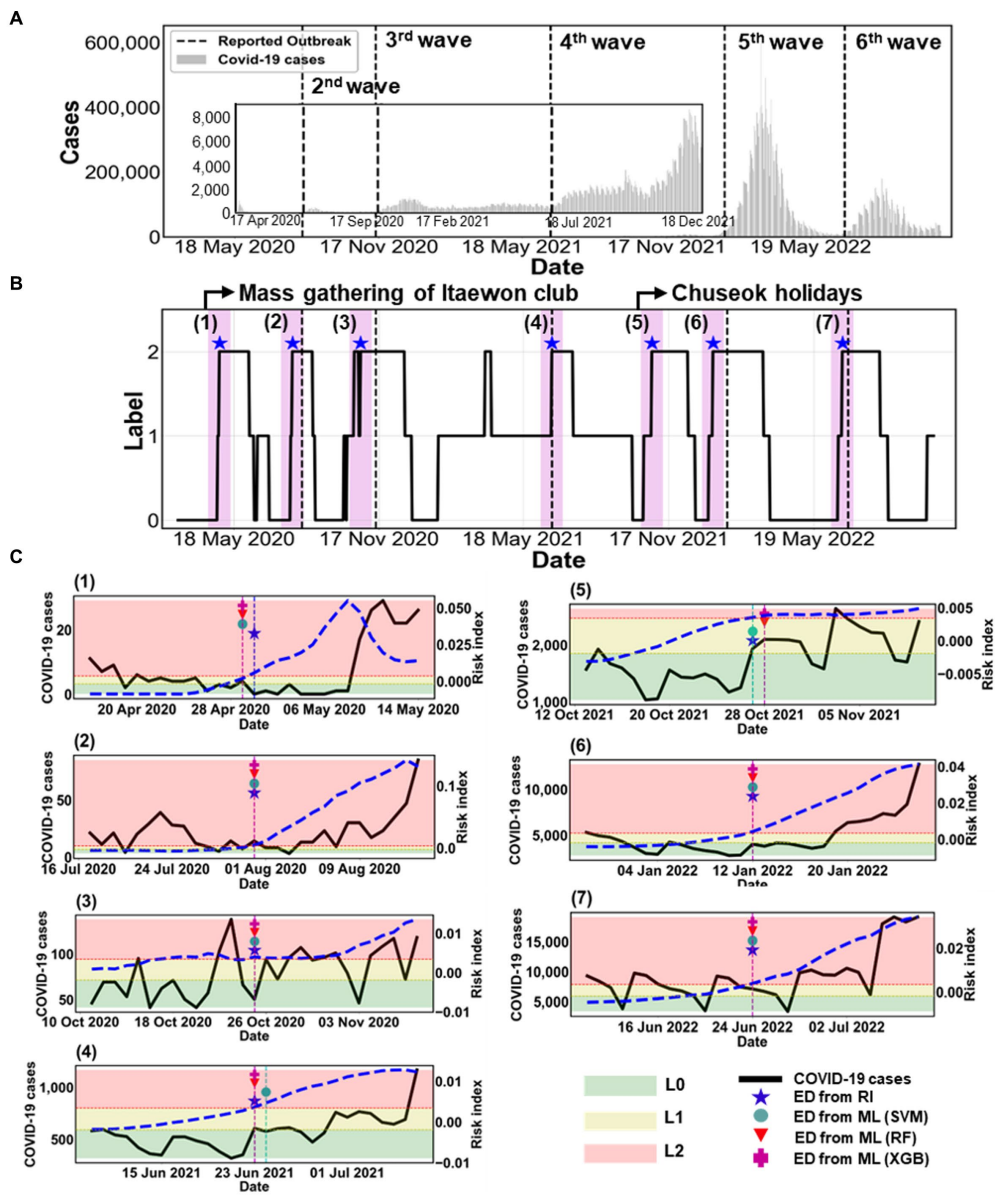
Comparison of estimated outbreaks. **(A)** The epidemic curve is shown from 18 February 2020 to 31 October 2022. The black dashed lines mark five reported outbreaks, described in Supplementary Table S5. **(B)** The label is obtained from the risk index in the black solid line. The blue asterisk (★) represents ED from RI. The magenta shaded region indicates the warning period from ED from RI. **(C)** Comparison between ED from RI and ED from ML during the warning period from ED from RI for (1)–(7) estimated outbreaks. The black solid line shows the number of COVID-19 cases (left *y*-axis). The blue dashed line shows the calculated RI on the right *y*-axis. ED from ML are marked as SVM (●), RF (▼), and XGB (+). The shaded areas indicate the labels as L0 (green), L1 (yellow), and L2 (red) according to the risk index.

Figure 6 summarizes all estimated outbreaks. Figure 6A displays the number of COVID-19 cases with the five reported outbreaks. We obtained seven estimated outbreaks, numbered (1)–(7), based on ED from RI in Figure 6B. Black dashed lines in Figure 6B indicate the reported outbreaks. This method declared the ED a few days earlier than the start time of reported outbreaks. There were seven estimated outbreaks, including the 1st and 5th ones [(1) and (5)], while there were only five reported outbreaks.

Figure 6C shows the specific results of each outbreak using ML methods. The figure also displays the COVID-19 cases (black solid line) and the risk index (blue dashed line). The ED from RI and the ED from ML predicted the same start dates of the (2), (3), (6), and (7) outbreaks. However, for the (1), (4), and (5) outbreaks, the ED from RI and the ED from ML differed by only 1 day. This means that both methods predicted almost identical start dates.

Table 3 summarizes the accuracy of the results between the reported and estimated outbreaks. We compared the accuracy of ML on the start time of outbreaks (1)–(7). We examine the results during the warning period, which was between 2 weeks before and after the ED from RI. The overall accuracy was high, ranging from 80% to 100%. Regarding the warning period for 4 weeks, RF showed the most accurate estimation with 100% accuracy, except for (1) and (5) outbreaks. This implies that RF detected the ED better for the rapid increase in a trend than other ML methods such as SVM and XGB.

**Table 3:** Comparison of the accuracy of the test data between the reported outbreak and estimation of ED using ML method (ED from ML).

	Reported outbreak^a^	ED from RI	ED from ML					
			ED from SVM		ED from RF		ED from XGB	
Estimated outbreak	Date	Date	Date	Accuracy	Date	Accuracy	Date	Accuracy
(1)	—	2020-04-30	2020-04-29	0.923	2020-04-29	0.923	2020-04-29	0.923
(2)	2020-08-12	2020-07-31	2020-07-31	0.857	2020-07-31	1.000	2020-07-31	1.000
(3)	2020-11-13	2020-10-25	2020-10-25	0.857	2020-10-25	1.000	2020-10-25	1.000
(4)	2021-06-23	2021-06-23	2021-06-24	0.889	2021-06-23	1.000	2021-06-23	1.000
(5)	-	2021-10-27	2021-10-27	1.000	2021-10-28	0.833	2021-10-28	0.833
(6)	2022–01–30	2022-01-12	2022-01-12	0.857	2022-01-12	1.000	2022-01-12	1.000
(7)	2022-07-01	2022-06-24	2022-06-24	1.000	2022-06-24	1.000	2022-06-24	1.000

There are seven outbreaks estimated from ML methods during a 4 weeks, denoted by (1)–(7). The date of ED from RI and ED from ML shows the timing of the early outbreak from the estimation.

a Reported outbreak represents the start time of the outbreaks, summarized in Supplementary Table S5.

Supplementary Figure S4 compares ED from RI with ED from ML by different durations of maintenance. When the duration changed to 7 or 21 days, there was no significant difference in the results. However, starting from 28 days, some outbreak detection points were not identified for a few outbreaks.

So far, we have used the training and testing datasets with a random 7:3 split ratio. Here, we conduct a simulation to assess the applicability of our approach for future prediction of the transmission trend. We divide the data into the train data from February 2020 to April 2022, when the omicron variant became prominent, and the test data from May to October 2022. We obtain sufficiently high accuracy on the test data as 0.8647 for RF and 0.8529 for XGB, even though those values decrease by approximately 5%–10%, compared to predictions made with randomly shuffled data. We need to figure out if our estimation can capture the fact that the start time of the 7th outbreak falls within the test data period.

**Figure 7:**
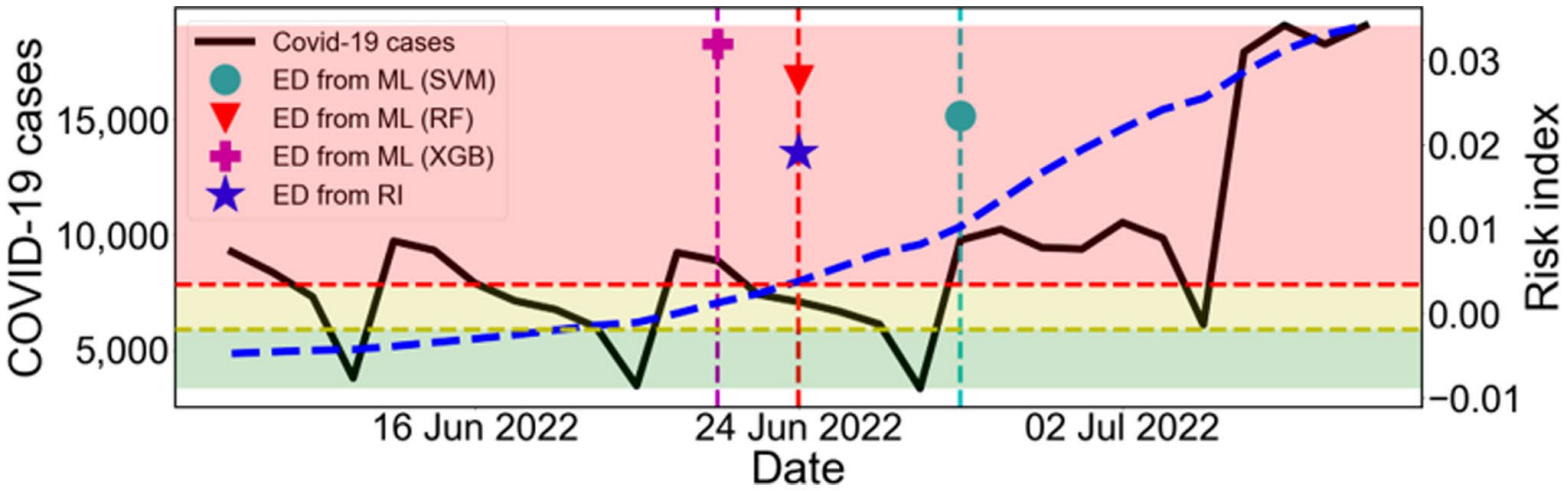
Estimation of the outbreak using the train data (February 2020–April 2022) and the test data (May 2022–October 2022). Comparison between ED from RI and ED from ML during the warning period from ED from RI. The black solid line shows the number of COVID-19 cases (left *y*-axis). The blue dashed line shows the calculated RI (right *y*-axis). The results of ED from ML are marked as SVM (●), RF (▼), and XGB (+). The shaded areas indicate the labels as L0 (green), L1 (yellow), and L2 (red) according to the risk index.

Figure 7 shows the result of the estimation using the train data (February 2020–April 2022) and the test data (May 2022–October 2022). Based on the ED from RI results, the start time of the 7th outbreak was determined to be on 24 June 2022. In comparison, the machine learning predictions yielded the following results: the ED from SVM and the ED from XGB were 4 days later and 2 days earlier, respectively. However, the ED from RF accurately predicted the exact same day. Therefore, this result confirms that our approach can effectively predict the early outbreaks.

## 4 Discussion

In the present study, we aimed to propose a machine learning-based method to predict the transmission trend of COVID-19 and to detect the start time of new outbreaks by analyzing epidemiological data in the Republic of Korea. To do so, we first, evaluated the performance of ML methods such as SVM, RF, and XGB in estimating the transmission trend. We developed a risk index to measure changes in the transmission trend, which were categorized into three groups: decrease (L0), maintain (L1), and increase (L2). We achieved a high accuracy (over 94%) in predicting the classification of transmission trends. Specifically, the SVM, RF, and XGB methods yielded accuracies of 0.9441, 0.9580, and 0.9545, respectively, as shown in Figure 3 and Table 2.

Second, we estimated new outbreaks from March 2020 to October 2022 in Korea. We proposed a new method for identifying the start time of new outbreaks when the label 2 is sustained for at least 14 days, which means the duration of maintenance is set to be 14 days. According to this standard, we estimated outbreaks using two approaches: (i) ED from RI, (ii) ED from ML. We obtained seven estimated outbreaks, numbered (1)–(7) based on ED from RI, as shown in Figure 6 and Table 3, while there were only five reported outbreaks. This means that the proposed method could be applied to detect minor outbreaks such as (1) and (5). We found that both the ED from RI and the ED from ML accurately predicted the same start dates for the (2), (3), (6), and (7) outbreaks. For the (1), (4), and (5) outbreaks, the ED from RI and the ED from ML differed by only 1 day. This indicates that both methods predicted start dates that were nearly identical. Additionally, we compared the accuracy of ED from ML in predicting the start time of outbreaks (1)–(7) during the warning period, which is the time period before and after 2 weeks from the ED from RI. The overall accuracy was high, ranging between 80%–100%. RF and XGB achieved the highest accuracy for outbreak detection, with 100% accuracy, except for the (1) and (5) outbreaks.

Third, we conducted a sensitivity analysis in our study, which included two components: (i) we evaluated the impact of different calibration periods (ranging from 14 to 28 days) and prediction periods (ranging from 7 to 21 days), with the calibration period being longer than the prediction period. Based on our analysis, we determined that the highest accuracy was obtained when using a calibration period of 21 days and a prediction period of 14 days, as presented in Supplementary Table S6. (ii) We varied the duration of maintenance for L2 between 7 and 28 days, as shown in Supplementary Figure S4. We observed that there was no significant difference in the results when the duration was changed to 7 or 21 days. However, when the duration was extended to 28 days, some outbreak detection points were missed for a few outbreaks.

This study has several limitations. First, previous studies (32, 33) have shown that vaccination reduces the number of severe cases. However, this study did not consider the effect of vaccination. We assumed that vaccination had a greater impact on reducing the number of infected patients than on the occurrence of outbreaks. Thus, we did not consider vaccination because we aimed to predict the occurrence and trend of outbreaks using classification methods.

Second, there is a limitation of insufficient data available, as COVID-19 has only had a period of 2 years of circulation compared to diseases such as influenza and norovirus that exhibit long-term epidemic patterns, which have been studied using ML to predict the start time of outbreaks in (34, 35). To overcome this, we analyzed the pattern of COVID-19 transmission in Korea and successfully extracted features that were highly related to the labels listed in Table 1. Consequently, we were able to achieve high accuracy in predicting the trend of epidemic patterns in three categories: increase, maintain, and decrease.

Despite these limitations, our study proposes a novel approach for estimating the start time of new outbreaks using machine learning methods and a risk index function, which has not been previously studied. Our approach offers several advantages and potential applications. In previous studies (14, 36), only the data on the number of infected patients were utilized for predictions of COVID-19 transmission. However, we incorporated various data, including the intensity changes in NPIs policies implemented by the Korean government and the prevalence of variant viruses (especially delta and omicron). Thus, our interpretation is comprehensive by analyzing the epidemiological data.

We newly suggested a risk index to quantify the changes of transmission trend. The risk index indicates the change of the transmission trend, which can be used to classify the risk of potential outbreaks. This measurement is a mathematically interpretable novel measurement that was not used in previous research. Using this metric, we are able to classify sample data into three distinct patterns (Increase, Maintain, Decrease) and assign labels accordingly.

Moreover, the variability in NPI intensity can be contingent on policy decisions. This means that by adjusting the NPI levels during the prediction period, we can anticipate shifts in future patterns of infection. This has the potential to assist in determining effective policy steps. In essence, our proposed predictive method can be utilized as a scientific foundation for establishing policy levels.

Previous research (14, 36) showed that the prediction accuracy for early detection of outbreak exhibited around 60%–80% even though the proposed methods were different. However, in the current study, employing machine learning techniques for the categorization on test data yielded a significantly higher accuracy of approximately 94%. Notably, a higher accuracy was achieved specifically for the Increase category (L2). By incorporating various datasets and utilizing the novel risk index for categorizing infection patterns, our proposed method contributed to achieving robust predictive performance even with limited data.

Overall, our study highlights the strength of our approach in accurately predicting the timing of an outbreak using an interpretable and explainable method. This method is also applicable to other infectious diseases and can contribute to the development of targeted prevention and control measures, facilitating better management of resources during the pandemic. It would enable healthcare providers to respond more effectively to COVID-19. Our proposed method identified outbreaks using machine learning-based approaches and can be further improved by collecting more data and establishing appropriate criteria for classes in future studies.

## 5 Conclusion

In conclusion, this study proposed a novel method for detecting the start time of new outbreaks and predicting transmission trends using machine learning-based approaches and a risk index function. The method achieved high accuracy in estimating the classification of transmission trends and successfully identified outbreaks with an interpretable and explainable method. The accuracy of SVM, RF, and XGB was higher than 0.94, with RF achieving the highest accuracy for outbreak detection. The method provides a standard for predicting the start time of new outbreaks, enabling healthcare providers to respond more effectively to COVID-19 transmission. Overall, the study demonstrates the strength of machine learning-based approaches in accurately predicting the timing of outbreaks, ultimately improving patient care and reducing the burden on healthcare systems.

## Data availability statement

The original contributions presented in the study are included in the article/Supplementary material, further inquiries can be directed to the corresponding author.

## Ethics statement

Ethical approval was not required for the study involving humans in accordance with the local legislation and institutional requirements.

## Author contributions

GC and JP: analyzed the data. GC, JP, YC, HA, and HL: drafted and revised the manuscript and interpreted the results. All authors contributed to the article and approved the submitted version.
