# Corrigendum: Detection of COVID-19 epidemic outbreak using machine learning

**DOI:** 10.3389/fpubh.2024.1381284

**Published:** 2024-02-22

**Authors:** Giphil Cho, Jeong Rye Park, Yongin Choi, Hyeonjeong Ahn, Hyojung Lee

**Affiliations:** ^1^Department of Artificial Intelligence and Software, Kangwon National University, Samcheok-si, Republic of Korea; ^2^Department of Mathematics, Kyungpook National University, Daegu, Republic of Korea; ^3^Busan Center for Medical Mathematics, National Institute for Mathematical Sciences, Daejeon, Republic of Korea; ^4^Department of Statistics, Kyungpook National University, Daegu, Republic of Korea

**Keywords:** COVID-19, prediction, machine learning, early detection, outbreak

In the published article, there was an error in the Funding statement. The correct Funding statement appears below.

