# Supplementary material for: Detection of COVID-19 epidemic outbreak using machine learning
Source: Front Public Health. 2023 Dec 18;11:1252357. doi: 10.3389/fpubh.2023.1252357 (PMC10764024; doi:10.3389/fpubh.2023.1252357)
Supplement: Supplementary file 1 [file Data_Sheet_1.pdf]

Supplementary Material

Detection of COVID-19 epidemic outbreak using machine learning

Giphil Cho[1,+], Jeong Rye Park[2,+], Yongin Choi[3], Hyeonjeong Ahn[4], Hyojung Lee[4,*]

*** Correspondence:** Hyojung Lee: hjlee@knu.ac.kr

Table S1. Reported outbreaks of the previous studies.

Reported outbreak	Start time	Sources
2nd wave	2020-08-12	[1-3]
	2020-07-28	[4]
	2020-08	[6]
	2020-08-16	[5]
	2020-08-27	[7]
3rd wave	2020-11-13	[1-3]
	2020-11	[6]
	2020-11-03	[4]
	2020-11-23	[5]
	2020-12-25	[7]
4th wave	2021-06-23	[1,3]
	2021-07	[6]
	2021-07-07	[2]
	2021-12-15	[7]
5th wave	2022-01-30	[2]
	2022-01	[1]
	2022-01-01	[3]
	2022-03-17	[7]
6th wave	2022-07-01	[1,3]
	2022-08-17	[7]

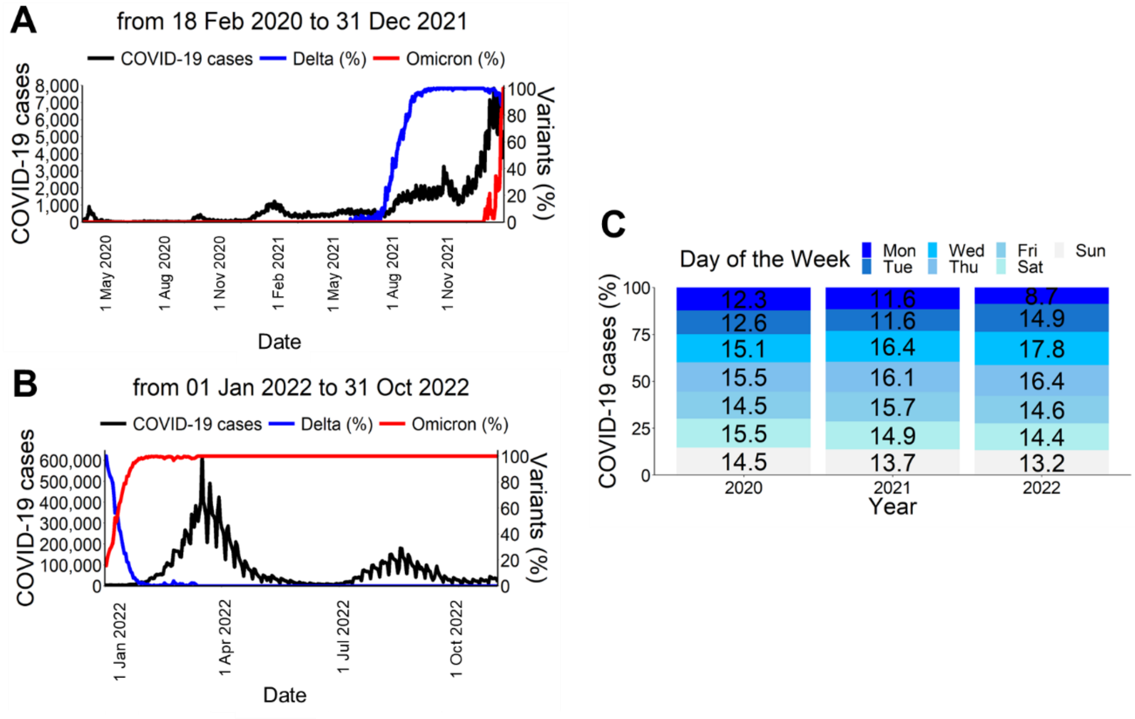

Figure S1. Transmission dynamics of the COVID-19 cases in Republic of Korea. Epidemic curve of reported cases and proportion of variants **A.** from 18 February 2020 to 31 December 2021 and **B.** from 01 January 2022 to 31 October 2022. **C.** The proportion of reported cases of COVID-19 by day of the week in 2020–2022.

Table S2. Summary of the important change of NPIs implemented in Seoul metropolitan area from Feb. 2020 to Oct. 2022

Date	Policy*	Description
2020-02-29 – 2020-03-21	2	The start of the social distancing in Korea [8]
2020-03-22 – 2020-04-19	3	Strong social distancing is implemented [9]
2020-04-20 – 2020-05-05	2	Relaxed social distancing is implemented [10]
2020-05-06 – 2020-08-15	1	Social distancing in everyday life
2020-08-16 – 2020-08-29	2	Enhanced social distancing as level 2 in Seoul metropolitan area
2020-08-30 – 2020-09-13	2.5	Enhanced level 2 is implemented<br>· Gatherings prohibited at academies (more than 10 people, less than 300 people), indoor sports facilities prohibited
2020-09-14 – 2020-10-11	2	Mitigation to level 2 in the Seoul metropolitan area<br>· Private education facilities (less than 300 people), lifting ban on gatherings at indoor sports facilities, making core quarantine rules mandatory, such as wearing masks
2020-10-12 – 2020-11-18	1	Adjustment to national level 1
2020-11-19 – 2020-11-23	1.5	Adjustment to level 1.5 in Seoul metropolitan area
2020-11-24 – 2020-12-07	2	Adjustment to Seoul metropolitan area level 2
2020-12-08 – 2021-02-14	2.5	Adjustment to Seoul metropolitan area level 2.5
2021-02-15 – 2021-07-11	2	Adjustment to Seoul metropolitan area level 2
2021-07-12 – 2021-10-31	4	New social distancing level 4
2021-11-01 – 2021-12-17	1	Step by step daily recovery (1 phase)
2021-12-18 – 2022-03-20	4	Outbreak contingency plan<br>· Private gatherings restriction: Private gatherings of 5 or more people are prohibited nationwide<br>· Other than private gatherings: Less than 50 people
2022-03-21 – 2022-04-03	3	Outbreak contingency plan extended<br>· Restriction on private gatherings: 6 people → 8 people
2022-04-04 – 2022-04-17	2	Outbreak contingency plan extended<br>· Restrictions on private gatherings: reduced from 8 to 10
2022-04-18 – 2022-10-31	1	Step by step daily recovery

Policy* indicates the feature describing the NPI levels implemented in Korea. KDCA provided a summary of NPIs implementations from May 2020 in [11].

Table S3. Number of reported cases of COVID-19 on the days of the week in 2020–2022.

	Mon	Tue	Wed	Thu	Fri	Sat	Sun
**2020**	6,821 (12.33%)	6,952 (12.56%)	8,344 (15.08%)	8,580 (15.51%)	8,015 (14.49%)	8,584 (15.51%)	8,036 (14.52%)
**2021**	64,972 (11.64%)	64,796 (11.61%)	91,417 (16.38%)	89,844 (16.10%)	87,664 (15.71%)	82,952 (14.87%)	76,345 (13.68%)
**2022**	2,172,781 (8.74%)	3,711,090 (14.92%)	4,427,342 (17.80%)	4,078,433 (16.40%)	3,634,220 (14.61%)	3,572,025 (14.36%)	3,274,745 (13.17%)

(·) represents the proportion of annual number of COVID-19 cases for each year from 2020 to 2022 in Korea.

Table S4. Range of parameters used for machine learning methods in the grid search process. Parameters are selected based on the highest accuracy.

**Methods**	**SVM**			**RF**		**XGB**	
	C	Gamma	Kernel	Number of trees	Maximum depth	Number of trees	Maximum depth
**Parameter** (Grid search range)	50 (10~200)	0.3 (0.1~1)	rbf (rbf, poly, sigmoid)	85 (50~100)	14 (1~30)	110 (5~200)	7 (1~20)

Table S5. Reported outbreak criterion of COVID-19 epidemic from 2020 to 2022.

Reported outbreak (Start time)	Description*	Main characteristics during the reported outbreak**
2nd wave (2020-08-12)	Outbreak mainly in the metropolitan area	Mass infection caused by assembly that was held at Gwanghwamun Square and gatherings in religious facilities
3rd wave (2020-11-13)	Outbreak mainly nationwide	Implementation of an NPIs to ban gatherings of five or more people
4th wave (2021-06-23)	Outbreak mainly due to Delta variant	Delta variant dominance
5th wave (2022-01-30)	Outbreak mainly due to Omicron variant	Omicron variant dominance
6th wave (2022-07-01)	Outbreak mainly due to Omicron variant	Omicron sub-variant (BA.5) dominance

* Detail description for ‘Description’ is provided in [1,2]
** Detail description for ‘Main characteristics during the reported outbreak’ is provided in [1,3]

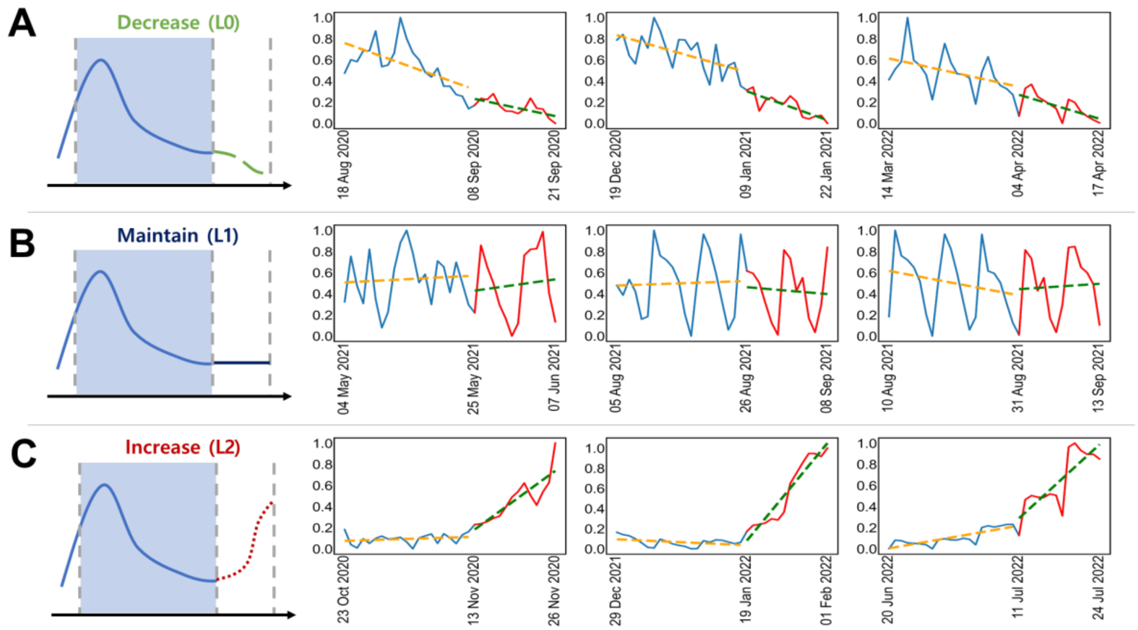

Figure S2. Examples of the sample data designated as labels. A. Decrease (L0), **B.** Maintain (L1), and **C.** Increase (L2).

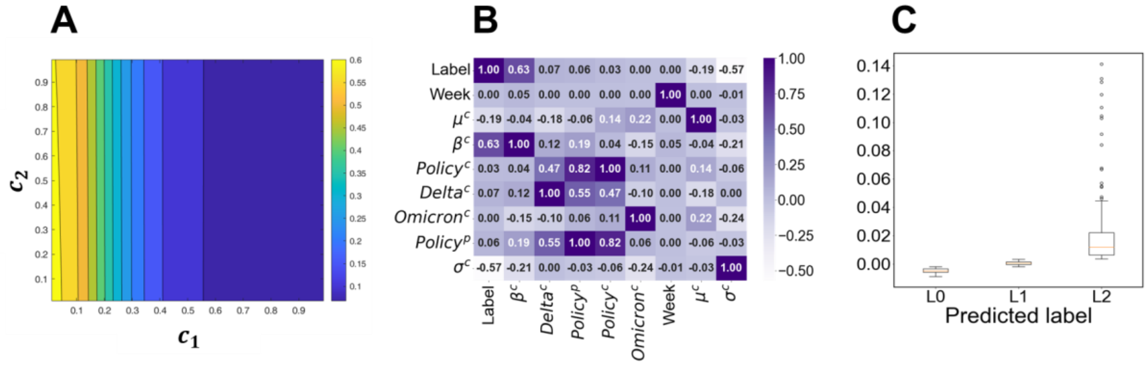

Figure S3. Scaling parameters of risk index and labels. A. Heatmap for scaling parameters of RI of Equation (1), **B.** Correlation of features and labels, **C.** Boxplot of risk index by labels for sample data.

Table S6. Average accuracy for test data by different calibration periods and prediction periods.

Calibration periods	Prediction periods		
	7 days	14 days	21 days
**14 days**	0.8744	-	-
**21 days**	0.9051	0.9522	-
**28 days**	0.9091	0.9214	0.9409

Average accuracy is computed for the different calibration periods and prediction periods, where we assume that the calibration periods are longer than prediction periods.

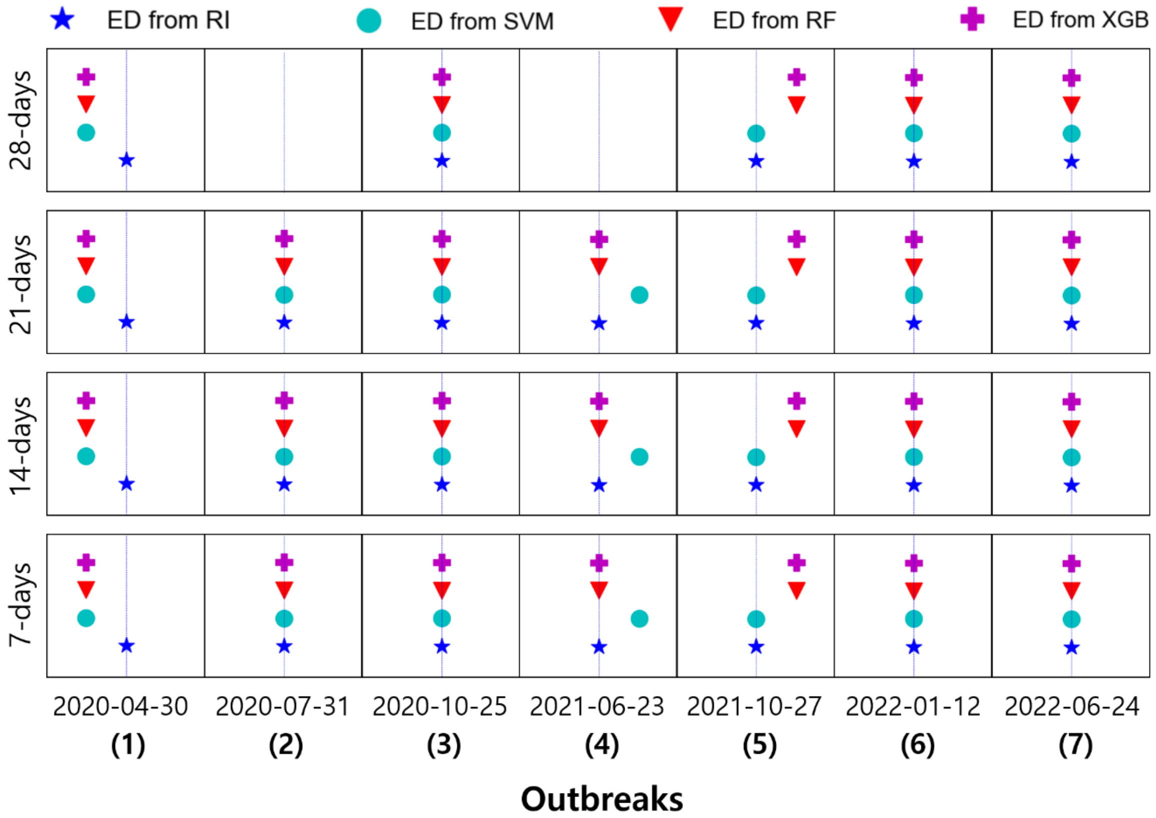

Figure S4. Comparison of estimated ED by different duration of maintenance. There are seven outbreaks we estimated. The blue asterisks (★) represents ED from RI, expressed with the dates. Estimation of start time of the new outbreak is compared by different duration of maintenance, such as 7-days, 14-days (baseline), 21-days, 28 days.

References

1. Hospital SNU. White paper of Seoul National University Hospital. 2022; Available from: http://www.snuh.org/m/board/B003/view.do?bbs_no=5963&searchWord.
2. Seonhee Ahn, et al. Outbreak report of COVID-19 during designation of class 1 infectious disease in the Republic of Korea (January 20, 2020 and April 24, 2022). *Public Health Weekly Report(PHWR),* 15(30), 2126-2136.
3. The weekly news review. Infectious Diseases Research Center of Seoul Metropolitan Government; 2022; Available from: https://sidrec.go.kr/index.do?contentId=76c6ce6e394bc21af6985a9d1b6610f8b0f7802d3814e8a38129d4e20982e97c&page=2.
4. Kim S, Kim M, Lee S, Lee YJ. Discovering spatiotemporal patterns of COVID-19 pandemic in South Korea. Sci Rep. 2021 Dec 28;11(1):24470. PMID: 34963690. doi: 10.1038/s41598-021-03487-2.
5. Lee JH, Park , Min S, Lee Sang WON. The Transmission Dynamics of SARS-CoV-2 by Setting in Three Waves in the Seoul Metropolitan Area in South Korea. Health and Social Welfare Review. 2021;41(2):7-26. doi: 10.15709/hswr.2021.41.2.7.
6. Kim D, Yoo TK, Lee Y, An M, An SE, Baek SJ. Changes of Inpatient Health Care Utilization in Public and Private Hospitals during the COVID-19 Pandemic. Health Insurance Review & Assessment Service Research. 2022;2(2):183-201. doi: 10.52937/hira.22.2.2.e10.
7. Jiu Lee SK. "Government says, the 7th wave in this winter... Up to 200,000 confirmed daily. The DONG-A ILBO; 2022; Available from: https://www.donga.com/news/Society/article/all/20221105/116325093/1.
8. Ha, J. H., Lee, J. Y., Choi, S. Y., & Park, S. K. (2023). COVID-19 waves and their characteristics in the Seoul metropolitan area (Jan 20, 2020-Aug 31, 2022). Public Health Weekly Report, 16(5), 111-36. doi: 10.56786/PHWR.20203.16.5.1
9. Ministry of Health and Welfare Press Releases Posted on 22 March 2020. Ministry of Health and Welfare; Available from: https://ncov.kdca.go.kr/tcmBoardView.do?brdId=&brdGubun=&dataGubun=&ncvContSeq=353673&contSeq=353673&board_id=&gubun=ALL.
10. Ministry of Health and Welfare Regular Briefing on COVID-19 Posted on 19 April 2020; Available from: https://ncov.kdca.go.kr/tcmBoardView.do?brdId=&brdGubun=&dataGubun=&ncvContSeq=354112&contSeq=354112&board_id=&gubun=ALL.
11. Public Data Portal, Republic of Korea; Available from [accessed 2023-08-20]: https://www.data.go.kr/data/15106451/fileData.do.
